# Context-induced contrast and assimilation effects in explicit and implicit measures of agency

**DOI:** 10.1038/s41598-019-40545-2

**Published:** 2019-03-07

**Authors:** Ke Ma, Bernhard Hommel, Hong Chen

**Affiliations:** 1 Key Laboratory of Personality and Cognition, Faculty of Psychology, Southwest University, Beibei, Chongqing, China; 2 Institute for Psychological Research &Leiden Institute for Brain and Cognition, Leiden University, Leiden, The Netherlands

## Abstract

Virtual-hand-illusion studies often use explicit and implicit measures of body ownership but no agreed-on implicit measure of agency exists. We investigated whether the Intentional Binding (IB) effect could serve as such a measure. A pilot study confirmed that current consistency increases both perceived agency and IB. In three experiments, current consistency was 50% but the previously experienced consistency was either 100% or 0%. When previous and present consistency experience were separated by a short break, both explicit judgments and IB showed a contrast effect. Eliminating the break reversed the effect in explicit agency but not in IB; and making the transition between previous and present consistency smoother replicated the effect for explicit agency but reversed the pattern for IB. Our findings suggest that explicit agency and IB rely on different sources of information, presumably including cross-sensory correlations, predictions of expected action-effects, and comparisons between present and previous consistency experiences.

## Introduction

Our body may be the object we know the best, as it is through the body that we interact with the outside world and experience it by generating exteroceptive and interoceptive sensory information. Usually people have no difficulty to differentiate their own body from those of others, but exactly how we cognitively represent our body and the actions it performs remains a mystery. Recent studies that aimed to shed some light on this mystery have made use of the notorious rubber hand illusion (RHI). When people are watching a rubber hand that is brushed/stroked synchronously, but not asynchronously, with their own unseen hand which is occluded from view (i.e., when the tactile and visual information matches), they tend to perceive the rubber hand as becoming a part of their own body^1^. This observation has been taken to reflect a *sense of body ownership* for the rubber hand, which is commonly distinguished from a *sense of agency*—the other component of what has been claimed to constitute the self^2^.

However, while some studies using the RHI have gathered evidence that explicit judgments of body ownership are unrelated to explicit judgments of agency^3,4^, some other studies using a variant of the RHI task that allows for voluntary action of the participant point to a rather tight relationship between body ownership and agency. In this variant, the rubber hand is replaced by a virtual hand, presented in virtual reality environment, which the participant can operate synchronously or asynchronously by means of a data glove^5^. Like with the RHI, operating the virtual hand without a temporal delay (i.e., synchronously) induces higher ratings of body ownership than operating it with a marked delay (i.e., asynchronously)—the virtual hand illusion (VHI)^6^. Interestingly, in the VHI paradigm, a very tight relationship (i.e., strong correlations) between perceived ownership and agency judgments can be observed^7^. This is likely to do with the fact that the virtual-hand design provides much more, and much more ecologically valid information than the rubber-hand design^8^: Feeling one’s own movement while seeing a virtual hand moving for a couple of seconds generates thousands of data points to compute visuomotor correlations. Visuomotor correlations are crucial for calibrating one’s senses, especially after dramatic changes or disturbances, as in mirror experiments^9^, which makes them a particularly obvious and ecologically valid source for both body ownership and agency. In the absence of such correlations, like in the classical rubber-hand setup, participants need to rely on other informational sources to generate body ownership and agency judgments. It makes sense to assume that these sources are more diverse^10^, less frequently used, and less rich and reliable—which would account for the observed discrepancy in the ownership-agency connection in RHI and VHI studies (for more information see^8^). While a recent review paper^11^ has focused on RHI and neglected evidence suggesting a strong ownership/agency association from VHI designs^7,8,12^, the authors do come to the conclusion that sense of ownership and agency interact, especially if voluntary action is allowed (in active RHI studies), suggesting that ownership and agency depend on overlapping information.

Of particular interest for the determination of the informational sources that might fuel ownership and agency are comparisons of explicit and implicit measures. Since Botvinick and Cohen (1998), explicit measures consist of ratings of the degree of perceived ownership and agency, but implicit measures are more diverse. To assess ownership, authors have often made use of proprioceptive drift rates (the degree to which the real, hidden hand is perceived to be close to the artificial hand) or skin conductance responses (the degree to which galvanic skin responses are shown when the artificial hand is under threat), but there is not yet any standard implicit measure for agency. The first major aim of the present study was to extend previous studies by testing the suitability of a measure that has often been treated as an implicit agency measure in studies unrelated to RHI and VHI: intentional binding (IB) (for reviews, see^13,14^).

The term IB (which unfortunately is less theoretically neutral than one would expect for the description of an empirical observation) refers to the finding that people subjectively compress the time interval between a voluntary action and an external sensory action effect, as compared to an involuntary movement and a comparable sensory stimulus^15^. IB effects are commonly measured by means of two widely used paradigms^15^. The first one is the Libet-style clock paradigm, in which participants watch a clock face and its pointer rotation while pressing a key and listening to a tone. Depending on the particular version of the design, their task can consist in judging the onset of several events: the participant’s own voluntary action, an involuntary action that the participant was made to execute, and the tone. The actual effect consists of two parts: the onset of the action is perceived later, and the onset of the tone is perceived earlier if the action is voluntary as compared to involuntary or absent. The common interpretation is that the causality between action and tone in the voluntary condition is somehow “binding” the tone to the action, which somehow creates the illusion that less time is passing between the two. The second classical task uses time interval estimation: here, participants directly estimate how long the interval between action and tone was and then vocally report the duration in milliseconds^16,17^. In this task, the IB is reflected by a shorter interval estimation if the action is voluntary as compared to involuntary or replaced by another event. The assumption is the same: the causality between action and tone creates the illusion that they occur closer in time.

The present study used a simpler IB task version but combined features of the two traditional IB tasks to test the assumption that IB represents a useful implicit measure of agency, as previously claimed^14,15^. Whereas explicit and implicit measures of body ownership are often assumed to differ with respect to the levels of representation involved (with implicit measures like proprioceptive drift being assumed to assess low-level multisensory integration rather than the high-level reasoning believed to be reflected by an explicit judgments), explicit agency perception and IB have been argued to be much more comparable. Indeed, IB has been claimed to be influenced by many factors including internal motor signals and external multisensory evidence about the source of actions and effects^16^, and to emerge from an interplay between pre-motor prediction and post-hoc reconstructive processes^17–19^, just like explicit agency does. If so, one would expect that IB should correlate with the standard explicit agency measure obtained by means of a questionnaire^20^, and it should be affected by experimental factors the same way as explicit agency is. Even though such effects have been demonstrated before with a RHI paradigm and a traditional (time interval estimation) IB task^21,22^, we deemed it essential to test whether they could be replicated with our VHI experiment setup and our modified IB task, as the validity of both tasks served as an important premise for our second experimental aim.

The second, and indeed more original major aim of the present study relates to the degree to which context can impact the perception of agency. According to strictly bottom-up approaches to self-representation along the lines of previous study^1^, one might expect that agency, just like ownership, is a function of particular contingencies between the movements one is carrying out and the perceivable effects they produce. However, recent studies of body ownership have indicated that perceived ownership is for instance affected by the previous familiarity with the artificial effector^23^, the similarity between real and artificial effector^24^, and by context-induced spatial reference frames^25^. This suggests a more interactive approach, according to which self-perception emerges from the interplay between bottom-up information and top-down expectancies^10,26^. Interestingly, contextual factors have also been reported to impact IB. Among other things, the size of the IB effect varies with repeated exposure to the interval separating action and delayed effect^27^, causal beliefs with regard to the manipulated action-effect^28^, or the cover story in which the IB assessment was embedded^29^. This is consistent with the suggestion that perceived agency can be accounted for by a multifactorial weighting process of different agency indicators^10^. It also fits with observations of tight relationships between ownership and agency senses with VHI designs^7,8,12^ and of ownership and agency being affected by experimental factors the same way^11^. Taken altogether, it is possible that ownership and agency may arise from at least partially overlapping informational sources, and so we were interested to see whether agency would be equally affected by context information than body ownership was in a previous study^25^. In particular, we were interested to see whether the context in form of previous experience with particular action-effect contingencies would affect explicit measures of agency and IB, the considered implicit measure of agency, and whether the context-induced effects would be comparable.

## Pilot Experiment

The pilot experiment used a well-established basic design^30^ to create a VHI. That is, participants wore a data glove that was hidden from their view and that operated a virtual hand on a screen in front of them. We manipulated what is commonly assumed to be the key variable to determine the degree of explicitly perceived agency — spatiotemporal consistency between the participant’s own real hand movement and the movement of the virtual hand (also called synchrony). Previous findings suggest that participants should show higher explicit agency judgments in the consistent than in the inconsistent condition^8^. As in previous RHI studies^21,22^ showing that, with active or robotic RHI, IB (estimated interval) was significantly larger in active congruent conditions than in active incongruent conditions, the interesting question for our pilot experiment was whether the implicit measure (the IB effect) would show the same effect and whether the size of this effect would correlate with the explicit measure of agency in our VHI design.

### Method

#### Participants

Twenty-two adults (6 male; mean age = 21.04, SD = 1.11, age range 19–24) from Southwest University, China, participated. All had normal or corrected to-normal vision, were naive with regard to the hypotheses of the experiment, and received payment for their participation. Participants gave their informed consent before the study, which was conducted in accordance with the ethical standards of the Declaration of Helsinki and with the ethical guidelines the local human research ethics committee at Southwest University, the methods were carried out in accordance with the relevant guidelines and regulations approved by the Research Ethical Committee of Southwest University (Chongqing, China).

#### Setup

**Figure 1:**
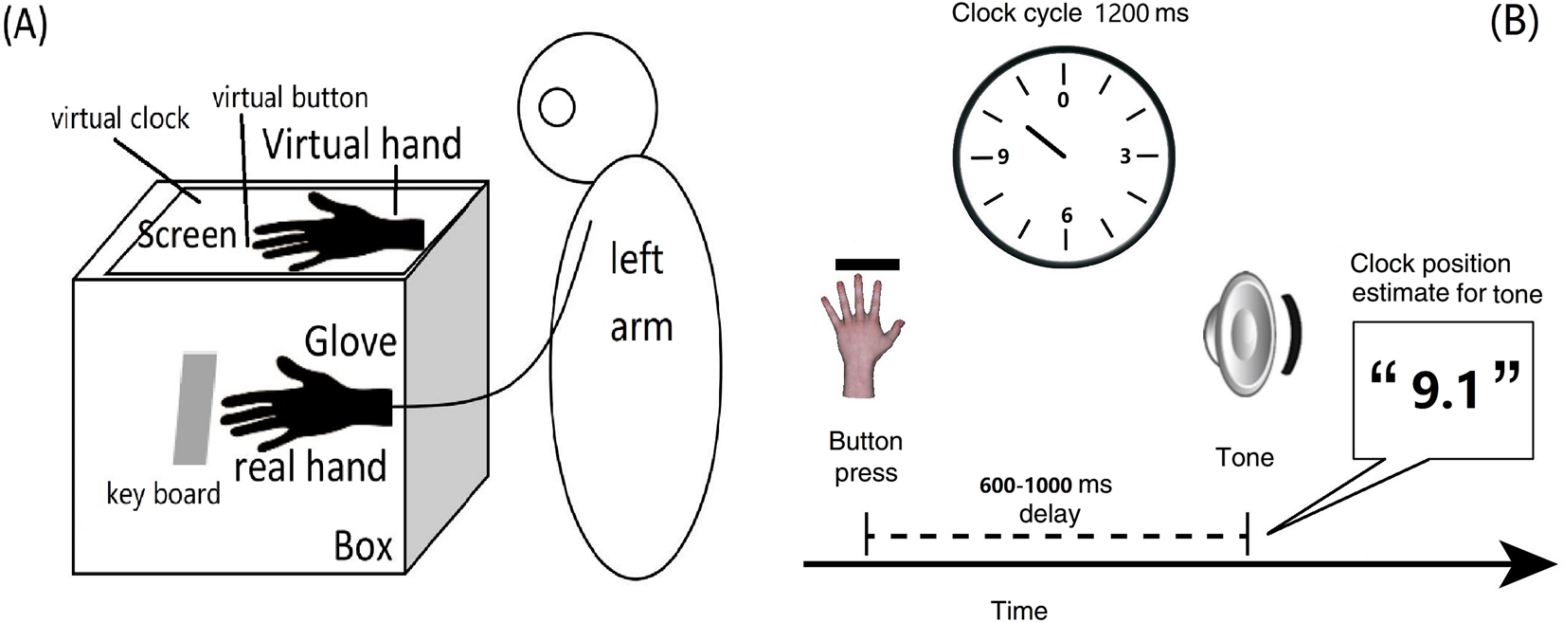
(**A**) The experimental setup, we omitted the virtual clock and virtual button, but only labeled their position on the screen during the time estimation task; (**B**) Illustration for one trial of the time estimation task.

The setup was similar as in a previous study^30^, as shown in Fig. 1(A), where a virtual hand served as artificial effector. We used a virtual reality environment/software (Vizard); a left-hand dataglove (5DT, measurement frequency = 75 Hz, latency = 13 ms) that participants wore on their left hand; a black box (width 54.4 cm × depth 23 cm × height 12 cm), in which the participant put his or her left hand along the depth axis, so to shield it from view; and a cape placed over the participant’s left shoulder to cover the space between the participant and the virtual effector. The virtual hand and dataglove module were imported into Vizard, so that the virtual hand that was shown in the middle of the computer screen on the box was controlled by the data from the dataglove, i.e., by the participant’s real hand movement, through script in the VR software in all conditions.

#### Design

We manipulated one within-participants factor—movement consistency, which represents the extent to which the movement of participants’ real hand controlled the movement of the virtual hand. There were two conditions, consistent and inconsistent. In the consistent condition, we simply transferred 100% of the real hand junction angle data collected by the data glove to the virtual hand through the VR script, so that the virtual hand movement was consistent with the real hand movement of the participant. In the inconsistent condition, we transferred random computer-generated noise (0% the real hand junction angle data) instead. To avoid possible coincidences of this noise and participants’ real hand movement, we designed the random virtual hand movement to be usually odd and trembled. Accordingly, the movement of the virtual hand was entirely unrelated to the movement of the real hand. For convenience, we use the labels 100% or 0% consistent to represent consistent and inconsistent conditions, respectively. However, note that the 100% label does not imply that the two movements were exactly the same as latency caused by the equipment was 13 ms. Hence, consistency (100%) is meant to imply that the transfer of real movement data to virtual movement data was unmodified/undistorted while the modification/distortion was maximal in inconsistent (0%) conditions. Participants were not informed about the degree of consistency. The sequence of the two conditions was fully counterbalanced across participants.

#### Procedure

When participants first came to the lab, they were asked to put on the data glove on their left hand, to put their left hand into the box, and to put on the cape. The cape was made in such a way that it covered the box and participant’s arm, so that participants could not see their real left hand but only watch the virtual hand on the computer screen. We also put a keyboard near to participants’ left hand, so they could press the space key with their real finger.

There were three phases in each of the two conditions. First, the virtual hand was shown on the screen, participants were asked to freely open and close their left hand, and watch the corresponding movement of the virtual hand for two minutes. The movement of the virtual hand is 100% or 0% consistent with the real hand movement. Second, a virtual button was shown near to the virtual hand finger, a virtual clock and its pointer were shown on the top right corner of the screen, participants were asked to perform the time estimation task which we described below. They were asked to voluntarily press the space key at their will with their real finger on the real keyboard. When the space key was pressed, the clock pointer started to rotate. Participants were to pay attention to both the virtual finger movement and its contact with the virtual button, and also the position of time pointer. When they heard a subsequent tone, they were to report the pointer position at tone occurrence. This phase contained ten identical trials, participants reported ten positions that represented the onset time points of the ten tones (for more information, please see below). Third, participants were asked to fill in the agency questionnaire. There was a 2-min break between each two conditions during which a “break” image was shown on the computer screen, so to reduce possible transfer effects.

Note that our procedure differed slightly from the two active RHI studies^21,22^ investigating the IB effect, in which participants only experienced the pressing and reporting phase. In our experiment, we asked participants to freely experience the consistent/inconsistent movement conditions for two minutes, before the pressing and reporting phase. The reason was that we wanted to maximize the agency-inducing experience through this phase.

#### Agency questionnaire

To assess the extent to which participants experienced agency (the explicit measure), we used an adapted Chinese version of the RHI/VHI questionnaire^1,5,31^. In particular, we presented participants with four questions assessing perceived agency sense (Q1–4), in which the most direct agency question Q1 and aggregated agency ratings^4^ are the most important indicators for agency. For each statement, participants responded by choosing a score on a 7-point (1–7) Likert scale, ranging from 1 for ‘strongly disagree’ to 7 for ‘strongly agree’. The statements were:

Q1. The movement of the virtual hand on the screen was caused by me.

Q2. I can control this virtual hand as I wish.

Q3. I can move my hand to control the virtual hand to contact the virtual button.

Q4. I can move my hand to control the virtual hand to contact the virtual button, and to cause the tone.

#### Time estimation

In line with an earlier study^15^, we simplified and combined features of the two traditional IB methods discussed above. We put the keyboard close to the other end of the black box, so participants could press the space key with their real left hand which stretched through the box without seeing the keyboard. To make the setup more ecologically valid, we designed a virtual button on the computer screen near the virtual finger; and also a virtual clock and a pointer that were shown on the top-right corner of the computer screen, far from the virtual hand and button.

We told participants that they could press the (unseen) space key whenever they wanted, pay attention to the clock pointer rotation, and estimate the time point when they heard a tone afterwards by reporting the position of the clock pointer. The clock face was marked with conventional intervals (0, 3, 6, 9), and the initial pointer position for each trial was always set to 0. Pointer rotation was initiated by the subject pressing the space key with their real left hand, and the clock pointer always rotated with a period of 1,200 ms for each trial, that is, with every press, the clock would run a full round, from 0 to 0. Participants were instructed to pay attention not only to the tone and the pointer position, but also to the virtual hand movement. Participants were asked to make fast and discrete keypressing movement to ensure that the tone-onset estimates could be easily identified. They were also encouraged to verbally report the time as precisely as possible (to one decimal place), not just the numbers shown on the clock face. See Fig. 1(B).

In the consistent movement condition, when participants pressed and released the space key with their real left-hand finger, they could see the same movement of the virtual hand pressing the virtual button, the virtual button went down and back just as the real space key. In the inconsistent movement condition, when participants pressed and released the space key, they did not see the exact same pressing movement on the screen but only random movement of the virtual hand; the virtual button went down and back, no matter whether the virtual hand contacted the virtual button or not. In a word, the only difference during the pressing action was the seen movement consistency between virtual hand and button. The tone and virtual button movement were both triggered by the contact between real hand and space key. The tone occurred at a time point that was set randomly between 600 and 1000 ms after the space key was pressed^20,21^, which was not told to the participants.

Each of the conditions consisted of ten identical trials, so we recorded ten time estimates that could be compared to the actual time for tone occurrence. For each trial, we subtracted the perceived onset time from the real onset time of the tone following the action, and divided it by the real onset time, then computed the mean for all trials—the average IB effect was defined as the average misestimation of the actual time in percentage^21^. Note that the time estimates difference should be relatively higher when the strong IB than weak IB occurs, which in turn have been taken to indicate stronger implicit agency. The reasons for using only ten instead of the more common 20–40 trials used in IB studies had to do with our rather realistic virtual-hand manipulation. In our virtual setup, participants could freely move the virtual hand by moving their own, which should induce a rather strong sense of agency. This strong experience could contrast with the very limited agency experience obtained during the rather passive time estimation task, where all activities were restricted to pressing a button. We were afraid that such a relatively passive situation might make the agency experience vanish soon. To reduce this possibility, we wanted to keep the time estimation short both in terms of trials and in terms of conditions, which is also why we preferred the shorter time estimation task over the clock or interval estimation paradigms. Second, empirical observations suggest that IB effects in the time estimation task are particularly strong in the earlier trials^32^, suggesting that having only few trials might not be a disadvantage. In any case, given that the pilot study and all following experiments (which we presented below) demonstrated reliable IB effects with ten trials, this demonstrated the suitability of our choice.

Overall, we note modifications of the original time estimation tasks, and this is why we call our method a simplified version of traditional IB tasks. First, we adopted the interval estimation paradigm^21,22^, as we did not want participants to vocally report at least two onset times (for the press action and the tone) for each trial as in the clock paradigm, which would increase our experiment time very much; Second, we presented a clock face and a pointer for participants as in the clock paradigm. The reason was that these other studies used practice trials for the time estimation task to familiarize participants with the task^21,22^—which would again increase the time spent on the rather passive estimation task and thus work against agency. By using the clock face and the pointer we provided an intelligible reference that made practice trials superfluous. Third, we set the pointer to rotate one round (from 0 to 0) after the participant pressed the key, while the tone occurred during 600–1000 ms after 0. In this way, participants could use this zero point as reference and only judge the tone onset time in relation to that (for the interval estimation), which should provide a clear reference that reduces other misestimation unrelated to the IB effect. In this sense, one can also think our simple IB task as an time point estimation task, as the onset time of the press action was always 0. As previously suggested^15^, with voluntary action the awareness of the following tone was shifted earlier toward the action, we assumed the tone onset time perception to be earlier in stronger IB than weaker or none IB effect conditions.

### Results

**Figure 2:**
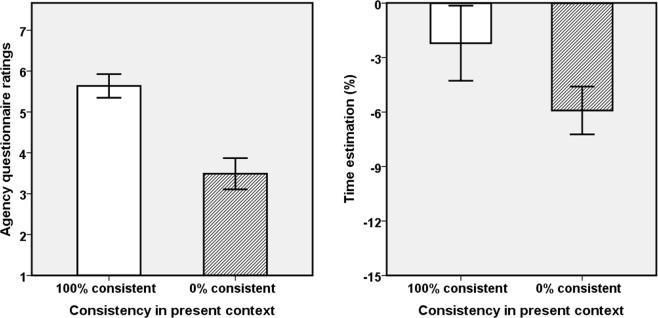
Explicit and implicit agency measures as a function of consistency. Left panel: Aggregated questionnaire scores for agency; Right panel: Time estimates. Error bars represent +/−1 SE.

We performed analyses with movement consistency (100% and 0%) in current context as within-subject independent variable, on the following dependent variables: agency (Q1–4) question ratings as explicit measurements, and time estimation task performance as implicit measurements for agency. See Table 1 and Fig. 2 for the results. A significance level of p < 0.05 was adopted for all tests. SE represents Standard Error. We also computed Spearman correlations between explicit and implicit results in the two consistent conditions but found no significant correlation (ps > 0.05, two-tailed).

**Table 1:** Means and standard errors (in parentheses) for the ratings of agency questions for both consistency conditions; F, P and Partial Eta squared (pη^2^)-values for the effects for all questionnaire items.

Consistency	Q1	Q2	Q3	Q4
100%	5.77 (0.29)	5.55 (0.33)	5.68 (0.29)	5.55 (0.35)
0%	3.05 (0.42)	3.18 (0.46)	3.95 (0.43)	3.77 (0.47)
F/P/pη^2^	25.58/<0.001/0.55	14.25/<0.001/0.40	14.30/0.001/0.41	12.11/0.002/0.37

#### Agency questionnaire

We ran both a repeated-measures ANOVA and a non-parametric analysis on the questionnaire data for aggregated agency (Q1–4). As they yielded the same results we only report the ANOVA results. The main effect of movement consistency was significant, *F*(1,21) = 20.04, *p* < 0.001, *pη*^2^ = 0.49, indicating greater perceived agency for the virtual hand when the movement of real and virtual hand were 100% consistent (mean = 5.64, SE = 0.29), than when they were 0% consistent (mean = 3.49, SE = 0.38), please see Fig. 2. We also analyzed the items separately and present the detailed data in Table 1. The scores of Q1, as the most direct agency question, were significantly different between the two consistent conditions.

#### Time estimation

The estimated time percentage were normally distributed, p(K-S test) > 0.05, so we ran a repeated-measures ANOVA. The main effect of movement consistency was significant, *F*(1,21) = 4.69, *p* = 0.042, *pη*^2^ = 0.18, indicating that participants estimated the onset time of tone as significantly earlier in time, and more toward to own action in the 100% consistency condition (mean = −2.21, SE = 2.07) than in the 0% consistency condition (mean = −5.91, SE = 1.32), which implied stronger implicit agency in the former than in the latter condition.

### Discussion

We manipulated movement consistency to see whether it would affect explicit and implicit measures of agency in comparable ways. For both explicit judgments and implicit perception of agency, significant main effects of movement consistency were found, suggesting that participants tended to perceive the virtual hand as being more controlled by themselves when it moved consistently with their real hand^33^. However, while explicit and implicit measures showed similar effects (with the shorter estimates suggesting a closer binding between action and outcome and, hence, more agency), these effects were not significantly correlated. This finding replicates earlier observations^21,22^. In any case, however, the pilot study confirmed that our experimental setup is suitable to evoke standard VHI effects and IB effects in both explicit and implicit measures of agency.

Interestingly, it seems that our participants tended to over-estimate the time between keypress and tone, while previous studies have often observed under-estimations^15,21,34^. One reason may be that in our VHI paradigm participants did not see their own hand touching the space key directly but saw the virtual hand moving and touching a virtual button. Given that these visual action effects were obviously more remote than those perceived in a standard IB study, participants might have taken longer transmission times into account, which may lead them to add extra time to their estimates. Another reason may be attributed to our modification of the time estimation task. The clock and pointer may reduce possible unrelated estimation errors but may conversely lead participants misjudge the tone as occurring with a delay. While this speculation calls for further research, the significant effects that we did find demonstrated that our design was sufficient to pick up IB-like effects in principle. Moreover, we will see that all experiments reported here showed comparable over-estimation tendencies on average, suggesting that this tendency is unrelated to, and cannot explain the other experimental effects we will be reporting.

For our interest, we run an additional short experiment for another 22 participants (2 male; mean age = 20.68, SD = 0.84, age range 19–22), in which we placed participants in the same experiment setup, but did not present them any clue about this experiment design and the virtual objects, only asked them to press key when they want. The results revealed that, the averaged time estimation percentage was −12.12%. This finding clearly demonstrated that the experiment setup caused participants to over-estimate the time for tone onset as we assumed, and the time estimation percentage for our two conditions in pilot experiment were both higher than this baseline time estimation, suggesting that IB effect was successfully induced. The fact, that both the 100% and 0% consistency would lead to IB effect, is consistent with previous studies^4,33^, with voluntary intention, even the 0% consistency may lead to a tendency to attribute authorship of actions to the self of participants. Note that all experiments reported in this article used a somewhat different design and tested different participants, so that we did not use the pilot data to subtract the baseline time estimation percentages (−12.12%) from the IB data—a common practice in previous IB studies^15^—but present it as a general reference only.

## Experiment 1

The basic rationale underlying our first experiment was derived by combining the insights from previous studies. For one, it is known that the perceived ownership of an artificial hand is affected by the spatial relationship between one’s real hand and the artificial hand. Perceived ownership is rather strong with no or minimal gaps between real and artificial hand^35,36^, but systematically declines as the distance between artificial and real hand increases^31,36^. However, an earlier study^25^ manipulated two kinds of previous context for the same test context and found evidence for a systematic impact of previous context: in the exact same condition (with the same distance between real and artificial hand in the same visuo-tactile stimulation), participants showed a stronger ownership illusion after having experienced a longer distance between real and artificial hand than after having experienced a shorter distance. This suggests that the impact of posture congruency does not rely on absolute but relative values—a so-called contrast effect. For another, we have previously argued, and empirically demonstrated that perceived ownership and agency are much tighter correlated in VHI than earlier RHI studies have suggested, which implies that ownership and agency rely on at least partially overlapping informational resources^8^. For instance, researchers manipulated exclusivity^33^, which was predicted to be a key criterion for perceived agency^37^, for illusory ownership perception. It was indeed observed that exclusivity affected agency and ownership alike, showing that the two measures may be influenced by same manipulations. If so, context, such as previous experience, should not only affect ownership but also agency as well. Accordingly, we tested participants in a condition with medium (50%) spatiotemporal consistency between their own movements and the movements of the virtual hand after having them exposed to either higher (100%) or lower (0%) consistency. To achieve the medium (50%) spatiotemporal consistency, we mixed 50% consistent data (data from real hand movement) and 50% inconsistent data (data generated by computer randomly). If spatiotemporal consistency would operate in a relative fashion, just as position congruency does^25^, this manipulation should generate more perceived agency in the post-inconsistent condition than in the post-consistent condition. Again, we are interested to investigate the relationship between explicit and implicit agency measures.

### Method

The method was as in the pilot experiment with the following exceptions.

#### Participants

Another twenty-eight adults (16 male; mean age = 20.32, SD = 1.25, age range 18–23) from Southwest University, China, participated.

#### Design

Experiment 1 was similar to the pilot experiment but employed the design from a previous study^25^. That is, we did not manipulate consistency in the current context (i.e., the degree of spatiotemporal consistency experience before being tested on agency) but consistency in the previous context, and investigated its influence on the same current context. While consistency in the current context was always 50%, previous consistency was either 100% or 0%. Participants were not informed about the degree of consistency and they were exposed to both previous consistency conditions before being tested under 50% consistency. The sequence of the two previous-consistency conditions was fully counterbalanced across participants.

#### Procedure

The procedure was similar to the pilot experiment, but with important changes. There were four phases in each of the two conditions. The first phase was the same as in the pilot experiment, in which participants freely moved their real left hand and watched the corresponding movement of the virtual hand for two minutes. The movement of the virtual hand was 100% or 0% consistent with the real hand movement. Next, participants were asked to take a half-minute rest during which the virtual hand disappeared from the screen. The third phase was similar to the first phase, in that the same virtual hand was shown on the screen and participants were free to move it with their own hand for another two minutes, the movement consistency was always 50%. Fourth, participants performed the same time estimation task and filled in the questionnaire as in pilot experiment. There was also a 2-min break between each two conditions.

### Results

**Figure 3:**
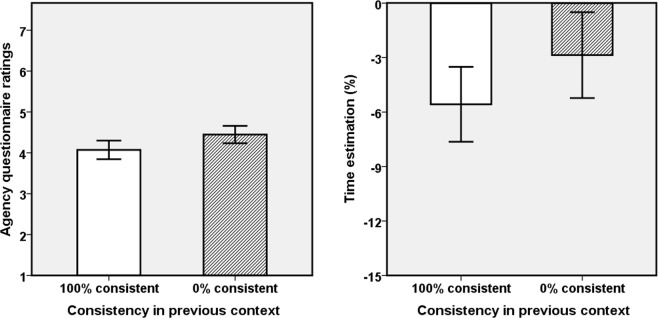
Explicit and implicit agency measures as a function of previous consistency. Left panel: Aggregated questionnaire scores for agency; Right panel: Time estimates. Error bars represent +/−1 SE.

We performed the same analysis as in the pilot experiment; see Table 2 and Fig. 3 for results. Note that the horizontal axis represents the consistency in the *previous* context. We again computed the correlations between explicit and implicit results in the two consistent conditions but found no significant correlation (ps > 0.23).

**Table 2:** Means and standard errors (in parentheses) for the agency ratings as a function of previous consistency; F, P and Partial Eta squared (pη^2^)-values for the effects for all questionnaire items.

Consistency	Q1	Q2	Q3	Q4
100% to 50%	3.64 (0.29)	3.82 (0.31)	4.68 (0.28)	4.14 (0.31)
0% to 50%	4.36 (0.31)	4.64 (0.32)	4.54 (0.20)	4.25 (0.29)
F/P/pη^2^	6.46/0.017/0.19	7.28/0.012/0.21	0.33/0.573/0.01	0.18/0.676/0.01

#### Agency questionnaire

Given that the repeated-measures ANOVA and the non-parametric analysis showed the same results, we only report the ANOVA results, with movement consistency (100% and 0%) in previous context as within-subject factor. The main effect of previous movement consistency was significant for aggregated agency (Q1–4), *F*(1,27) = 4.82, *p* = 0.037, *pη*^2^ = 0.15, indicating that participants perceived stronger agency over the virtual hand after having been exposed to the 0% consistency condition (mean = 4.45, SE = 0.21) than after having experienced 100% consistency (mean = 4.07, SE = 0.23), as shown in Fig. 3. See Table 2 for more details and the outcomes of the separate item analyses. The scores of Q1, as the most direct agency question, were significantly different between the two consistent conditions. Note that, in contrast to the pilot study, only two of the four items produced significant results.

#### Time estimation

The estimated time percentages were normally distributed, p(K-S test) > 0.05, so we ran a repeated-measures ANOVA. The main effect of previous movement consistency was significant, *F*(1,27) = 4.74, *p* = 0.038, *pη*^2^ = 0.15, indicating more pronounced IB (time compression) after a 0% consistency context (mean = −2.86, SE = 2.36) than after a 100% consistency context (mean = −5.57, SE = 2.06), which in turn implies stronger implicit agency if previous consistency was 0%.

### Discussion

We manipulated previous movement consistency to see how that might affect perceived agency in the current, medium-consistent context, and whether it would affect explicit and implicit measures of agency in comparable ways. Both questionnaire ratings and time estimations showed evidence of stronger agency in a medium-consistency condition after participants had experienced a purely random relationship between their own movements and that of the virtual hand, than after they had experienced complete consistency. The results support our assumption that consistency in a previous context moderate perceived agency in the current context. Both explicit and implicit agency measures were moderated in similar ways, which fits with the previous observations of spatial context effects on body ownership^25^. These findings have a couple of interesting theoretical implications. First, they are consistent with our claim of a tight relationship between ownership and agency^8^, again corroborating that the two processes may rely on overlapping sources of information. Second, our findings fit with the assumption that perceived agency is not a direct function of particular consistency parameters or values but rather emerges from an interaction between these parameters and particular expectations: If participants expect (or are used to) a total lack of control over the virtual hand, as after previous inconsistency, medium control is perceived as an indicator of substantially more control but if they expect (or are used to) complete control, as after previous consistency, medium control rather seems to indicate little control. A similar observation was made by other researchers^38^, who investigated the impact of the delay between a self-performed action and an external action effect on perceived agency. While earlier studies had suggested that perceived agency decreases with increasing action-effect delay, they demonstrated that it is the expectation or adaptation^27^ that counts: if participants are used to zero delays, agency indeed decreases with increasing delay; but if participants were led to expect longer delays, perceived agency was most pronounced when the action effect occurred at the expected time point and decreased with increasing distance to this time point.

## Experiment 2

Experiment 1 provided evidence for a contrast effect of the previous consistency context on perceived agency in the current context. While this observation fits with earlier finding^25^ showing that the previous spatial consistency context affects perceived body ownership in the current context, it seemed to contradict a recent finding by Liepelt *et al*.^23^. These authors showed that perceived body ownership for a non-corporeal object is stronger if the person has a history of personal agency with this object, such as with a smart phone as compared to a wooden block. The authors only tested for body ownership effects but if a similar pattern would be obtained for agency, which our assumption of a tight ownership-agency relationship implies, this pattern arguably contradicts our present observations in implying that a high degree of control in a previous context increases, rather than decreases, ownership and, probably, agency. In other words, Liepelt *et al*. found assimilation between previous and present context while we found contrast.

It is true that many details of the Liepelt *et al*. study were very different from the present one, including the use of RHI in the former and VHI in the latter. But more important might be the temporal relationship between previous and present context. Operating a smart phone or a computer mouse, which was also studied in the same study^23^, is a very common activity in the present culture and thus unlikely to be bound to a particular situation or event. Accordingly, people are likely to represent a smart phone or a mouse as something that is rather context-independent, which would facilitate the generalization of previous experience. Encountering a smart phone or mouse would thus not only retrieve previous experiences but these experiences would also be easily integrated with a new situation. This is different from the present Experiment 1, where participants were exposed to a novel situation in a novel setup, together with a particular control experience, which was followed by another novel situation, together with a very different control experience. These experiences, and the contrast between them, are likely to lead to two different and easy discriminable event representations, and it may be this difference that induced the contrast, rather than assimilation. If these considerations are valid, it should be possible to turn the contrast effect observed in Experiment 1 into an assimilation effect by reducing the discriminability and, thus, the discontinuity between previous and present context. According to the event theory of Zacks and colleagues^39^, perceived event boundaries can be experimentally manipulated by changing the temporal contiguity of events, suggesting that the segmentation/discrimination of succeeding events (in our studies the previous and present experience) can be manipulated by changing the temporal discontinuity on possible boundaries. Accordingly, we conducted Experiment 2, where we tried to achieve that by replicating Experiment 1 except that we eliminated the half-minute rest between previous and present context in each condition. We hypothesized that this would make our findings more comparable to those findings^23^, in the sense that 100% consistency in the previous context would increase current agency while 0% consistency in the previous context should reduce perceived agency.

### Method

The method was as in Experiment 1 with the following exceptions.

#### Participants

Another twenty-eight adults (4 male; mean age = 20.86, SD = 1.35, age range 19–25) from Southwest University, China, participated.

#### Design and Procedure

Design and procedure were almost the same as in Experiment 1, but with only one change: The half-minute rest time between previous and present context in each condition was dropped, so that participants experienced a 100% or 0% consistent virtual hand before controllability suddenly changed to 50%.

### Results

**Figure 4:**
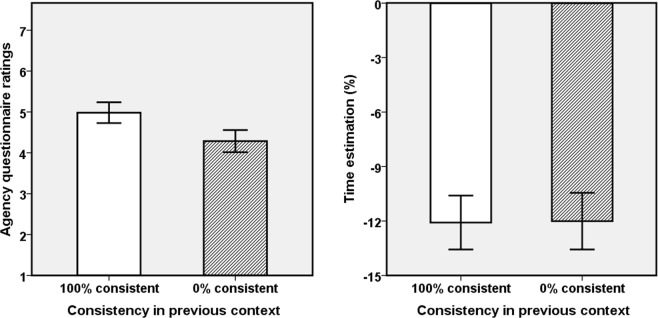
Explicit and implicit agency measures as a function of consistency between movement in previous context. Left panel: Aggregated questionnaire scores for agency; Right panel: Time estimates. Error bars represent +/−1 SE.

We performed the same analyses as in the pilot experiment, see Table 3 and Fig. 4 for results. We again found no significant correlations between explicit and implicit agency measures, ps > 0.11.

**Table 3:** Means and standard errors (in parentheses) for the agency ratings as a function of previous consistency; F, P and Partial Eta squared (pη^2^)-values for the effects for all questionnaire items.

Consistency	Q1	Q2	Q3	Q4
100% to 50%	5.43 (0.27)	4.86 (0.33)	4.96 (0.31)	4.68 (0.31)
0% to 50%	4.00 (0.35)	4.07 (0.34)	4.64 (0.34)	4.43 (0.35)
F/P/pη^2^	16.27/<0.001/0.38	3.74/0.064/0.12	1.30/0.264/0.05	1.34/0.257/0.05

#### Agency questionnaire

As the repeated-measures ANOVA and the non-parametric analysis yielded the same outcome, we report results for the former only. The main effect of previous movement consistency was highly significant for aggregated agency (Q1–4), *F*(1,27) = 9.39, *p* = 0.005, *pη*^2^ = 0.26, showing that participants perceived stronger agency after having been exposed to 100% consistency (mean = 4.98, SE = 0.25) than after having been exposed to 0% consistency (mean = 4.29, SE = 0.27), as shown in Fig. 4. Results from more detailed analyses for each item are presented in Table 3. The scores of Q1, as the most direct agency question, were significantly different between the two consistent conditions.

#### Time estimation

The estimated time percentages were normally distributed, p(K-S test) > 0.05, so we ran a repeated-measures ANOVA. The main effect of previous movement consistency was not significant, *F*(1,27) = 0.003, *p* = 0.96, *pη*^2^ < 0.001, indicating no significant IB effect difference between the two conditions (mean = −12.09, SE = 1.48 after 100% consistent vs. mean = −12.01, SE = 1.56 after 0% consistent context).

#### Discussion

Experiment 2 aimed to test whether eliminating the time break between previous and current consistency context would make the findings more comparable to those reported in an earlier study^23^, which basically would imply a reversal of the outcome pattern obtained in Experiment 1. This prediction held for the explicit agency ratings, which indeed reversed in the present experiment. This supported our speculation that, despite all further differences between our current setup and that used by Liepelt *et al*., it might be the relationship/continuity between present and previous experience that determines the direction of the effect. More specifically, a weak relationship/continuity seems to induce a contrast effect, as obtained in Experiment 1, while a strong relationship/continuity seems to induce assimilation, as observed in this experiment. We tried to strengthen the relationship/continuity by eliminating the break between previous and present contingency experience, but it is obvious that time cannot be the only factor: after all, Liepelt *et al*. found assimilation for objects that people had used in the not much more distant past^23^ but, as we argue, in so many contexts that the contingency experience generalized and thus became independent of the context. In any case, our findings provide preliminary evidence that linking previous and present contingency experience might promote assimilation while separating previous and present contingency experience induces contrast.

Interestingly, our prediction held for the explicit measure only, while the IB was basically unaffected by the manipulation. That is, eliminating the temporal gap between previous and present contingency experience reversed explicit agency but had no impact on IB. This suggests that explicit agency ratings and the IB are not based on the same information—an issue we investigated in Experiment 3.

## Experiment 3

The outcome of Experiment 2 suggests that our explicit agency measure is sensitive to manipulations that affect the perceived similarity/continuity between previous and present task context while our implicit measure, IB, is not. This might be because IB, or perhaps any implicit measure, is insensitive to cognitive manipulations in principle, perhaps because it relies more strongly on low-level multisensory information than explicit agency does. However, as pointed out in the introduction, IB has been demonstrated to be impacted by contextual, rather high-level factors in various studies, which renders this possibility unlikely. Another possibility could be that the sudden changes in consistency, which were still present in Experiment 2, prevented IB from showing an assimilation-like effect. Sudden changes of the current context, and the relatively dramatic failures in prediction that such changes imply, have been claimed to trigger a reset of working memory, which in turn leads to segmentation between succeeding events^39,40^. Magliano and Zacks have shown that segmentation of (or discrimination between) succeeding events can be affected by temporal and other kinds of discontinuities at possible event boundaries, and action-related discontinuities had a particularly strong impact^40^. Thus, to achieve situational continuity between previous and present experience and induce possible assimilation effect, action continuity (eliminating the sudden change) may contribute more than the temporal continuity (eliminating the time break), and IB may be more sensitive to action continuity than explicit agency is—perhaps because explicit agency relies more on high-level reconstructive information^10^. This scenario might not only account for the dissociation between agency ratings and IB observed in Experiment 2, but it also suggests an obvious prediction: making the transition between previous consistency or inconsistency to 50% consistency smoother should also reverse the IB pattern, which we therefore predicted to follow the explicit measure in Experiment 3.

### Method

The method was as in Experiment 2 with the following exceptions.

#### Participants

Another twenty-eight adults (4 male; mean age = 20.86, SD = 1.35, age range 19–25) from Southwest University, China, participated.

#### Design and Procedure

The procedure was almost the same as in Experiment 2, with only one change: consistency did not change suddenly but gradually, in a stepwise fashion, from 100% or 0% to 50%. Specifically, after 1.5 minutes of “control” experience with the 100% or 0% consistent virtual hand, movement consistency gradually changed to 50% over time. The entire transition took 1 min to finish, so that from then on participants experienced the 50% consistent virtual hand for 1.5 minutes. The transferred data to virtual hand from real hand movement decreased 0.83%, and the transferred data to virtual hand from computer-generated noise increased 0.83% every second for the 100% change to 50% consisent condition, for example.

### Results

We performed the same analyses as in the pilot experiment, see Table 3 and Fig. 4 for results. We again found no significant correlations between explicit and implicit agency measures, ps > 0.13.

#### Agency questionnaire

**Figure 5:**
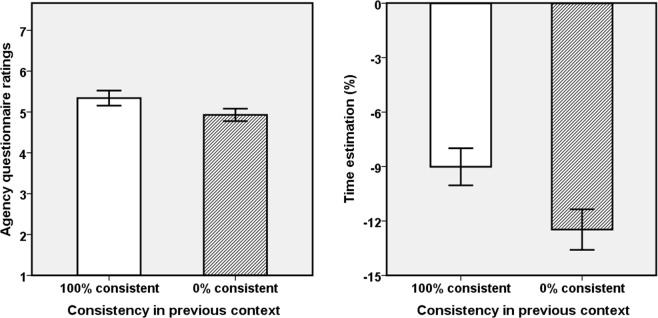
Explicit and implicit agency measures as a function of consistency between movement in previous context. Left panel: Aggregated questionnaire scores for agency; Right panel: Time estimates. Error bars represent +/−1 SE.

As the repeated-measures ANOVA and the non-parametric analysis yielded the same outcome, we report results for the former only. The main effect of previous movement consistency was significant for aggregated agency (Q1–4), *F*(1,27) = 8.15, *p* = 0.008, *pη*^2^ = 0.23, indicating that participants perceived stronger agency after having been exposed to 100% consistency (mean = 5.34, SE = 0.18) than after having been exposed to 0% consistency (mean = 4.93, SE = 0.15), as shown in Fig. 5. Results from more detailed analyses for each item are presented in Table 4. The scores of Q1, as the most direct agency question, were significantly different between the two consistent conditions.

**Table 4:** Means and standard errors (in parentheses) for the agency ratings as a function of previous consistency; F, P and Partial Eta squared (pη2)-values for the effects for all questionnaire items.

Consistency	Q1	Q2	Q3	Q4
100% to 50%	5.25 (0.26)	5.07 (0.26)	6.00 (0.23)	5.04 (0.36)
0% to 50%	4.50 (0.23)	4.64 (0.20)	5.64 (0.24)	4.93 (0.34)
F/P/pη^2^	7.99/0.009/0.23	2.84/0.103/0.10	4.72/0.039/0.15	0.21/0.648/0.01

#### Time estimation

The estimated time percentages were normally distributed, p(K-S test) > 0.05, so we ran a repeated-measures ANOVA. The main effect of previous movement consistency was significant, *F*(1,27) = 5.36, *p* = 0.028, *pη*^2^ = 0.17, indicating that participants estimated the onset time of the tone as significantly earlier in time and more toward to own action in the current context after having experienced the 100% consistent context (mean = −9.02, SE = 1.32) than after having experienced the 0% consistent context (mean = −12.48, SE = 1.57).

#### Discussion

The results confirmed our expectations, in the sense that we replicated the assimilation pattern obtained in Experiment 2 for explicit ratings and reversed this pattern for IB. This suggests that IB was sensitive to our gradual-change manipulation while explicit ratings were not—here the effect was numerically even slightly smaller than in Experiment 2. We can thus conclude that previous consistency experience impacts both explicit and implicit agency indicators but it does so in different ways and through different kinds of informational cues.

## Comparison ACROSS Experiments 1–3

Given that we had equal sample sizes for the three experiments and their designs were very similar, we ran analyses comparing the outcomes directly. Explicit agency ratings were analyzed by means of a 3 (experiment) × 2 (consistent vs. inconsistent) ANOVA, with experiment (1, 2 and 3) as between- and movement consistency (100% and 0%) in previous context as within-subject factor. The significant main effect of experiment, *F*(2,81) = 4.80, *p* = 0.011, *pη*^2^ = 0.11, was due to that the agency ratings varied across experiments, being highest in Experiment 3 and lowest in Experiment 1; and the significant consistency effect, *F*(1,81) = 5.28, *p* = 0.024, *pη*^2^ = 0.61, reflected the greater perceived agency in the previously-consistent conditions. Given that the interaction effect was also significant, *F*(2,81) = 9.10, *p* < 0.001, *pη*^2^ = 0.18, we calculated consistency effects ([agency ratings after previous consistency − ratings after previous inconsistency]/ratings after previous inconsistency) and then compared the effects by means of two-tailed independent t test across experiments. The effect differed between Experiment 1 and 2, *t*(54) = 3.44, *p* = 0.001, d = 0.99; and between Experiment 1 and 3, *t*(54) = 3.28, *p* = 0.002, d = 0.88; but not between Experiment 2 and 3, *t*(54) = 1.71, *p* = 0.097. These results are consistent with the scenario that eliminating the temporal break had an impact on explicit agency.

The IB effects were analyzed the same way. For the time estimation percentage, the main effect of experiment was significant, *F*(2,81) = 7.60, *p* = 0.001, *pη*^2^ = 0.16, while the main effect of consistency was not, *F*(1,81) = 0.79, *p* = 0.78. As the interaction was again significant, *F*(2,81) = 5.00, *p* = 0.009, *pη*^2^ = 0.11, we further analyzed the consistency effects (IB after previous consistency − IB after previous inconsistency) on IB. Comparisons revealed no difference between Experiment 1 and 2, *t*(54) = 1.40, *p* = 0.166; and no effect between Experiment 2 and 3, *t*(54) = 1.73, *p* = 0.09; but significant differences between Experiment 1 and 3, *t*(54) = 3.17, *p* = 0.002, d = 0.85. These results are consistent with this scenario that IB was affected by the elimination of sudden changes but not by the temporal break.

## General Discussion

The major aims of the present study were to test the suitability of IB as an implicit measure of agency with VHI design, and to investigate the role of previous agency-related experience on the perception of current agency. With respect to the first aim, the outcome is mixed, at least at first sight. On the one hand, we were able to show that explicit agency ratings and IB are affected by the same experimental manipulations and in the same way. This might be taken to imply that IB is a suitable implicit measure of agency, as claimed by various authors^13,14^. On the other hand, however, we also found evidence of clear empirical dissociations between explicit ratings and IB, such as the differential impact of the elimination of a temporal separation between previous and present experience in Experiment 2 and of the gradual transition introduced in Experiment 3. Moreover, we did not find a single significant correlation between explicit agency ratings and IB. Comparable dissociations between explicit and implicit measures has also been reported^41,42^, suggesting that it is a replicable phenomenon. Of interest, explicit and implicit measures of perceived body ownership have also been found to be uncorrelated in several studies^8,23,43^. Taken altogether, there is evidence that the IB is somehow related to agency but cannot be assumed to reflect the same information that explicit judgments assess.

We suggest that a constructivist perspective renders these observations much less problematic or contradictory than they may seem. If one considers both explicit and implicit agency measures as (results of) simple readouts of some internal code that represents subjective or objective agency, then any dissociation necessarily implies that those readouts did not target the same code. This in turn would raise the question which measure reflects “real agency”, i.e., which is the readout of the relevant code, and it would draw into doubt the validity of all measures that apparently reflect other kinds of information. But we see no good reason why such a code should exist. It is true that the organization of many societies relies on ownership and personal responsibility (an issue that touches personal agency), and many legal systems are based on aspects of the agency concept^44^ or at least illusions thereof^37^. While this societal development makes agency judgments by oneself and others increasingly important, the phylogenetic development of the human brain is much older than those more recent societal achievements, which renders it rather unlikely that a dedicated brain mechanism has evolved to compute agency. If so, people need to construct agency judgments ad hoc, based on some informational sources. There is no reason to assume that these informational sources need to be the same all the time, neither for the same individual nor for different individuals, and there is no reason to assume that all sources are relevant for all possible explicit and implicit measures of agency. The key question thus is what informational resources are available and feed into explicit agency judgments and implicit measures, like IB. Given the present outcome pattern, we consider three such sources.

First, intersensory correlations have been considered to represent an important basis of explicit judgments of body ownership since the original article on the RHI^1^. As we have argued in more detail elsewhere^8^, the observation that agency and ownership have sometimes been dissociated in rubber-hand studies but are strongly correlated in virtual-hand setups suggests that active movement of participants is a game changer with respect to the agency-ownership relation. Given that active movement potentiates the data points available for computing cross-sensory correlations, this suggests that perceived (explicit) ownership and agency rely on such correlations. If so, one would expect that the degree of the *present* consistency in current context between one’s own movements and those of a visible artificial effector predicts explicit agency ratings, which is indeed what we observed in the pilot study. We believe that present consistency affected explicit ratings in all other experiments as well. Given that present consistency (and thus the degree of cross-sensory correlation) was fixed in all other experiments, there cannot be any differential effect. Indeed, the mean rating scores obtained in Experiments 1–3 all fall in between the two more extreme scores obtained for the 100% and the 0% conditions in the pilot study. This fits the fact that present consistency was always 50%. However, there were differences in the explicit rating scores among Experiments 1–3, suggesting the role of different previous consistency context besides intersensory correlations in explicit agency perception, an issue we will get back to.

Second, another source that has been favored by many authors is prediction or, more precisely, the degree to which predictions are met (for an overview, see^45^). Wegner has suggested that unconscious processes of action selection generate predictions of to-be-expected sensory consequences of the currently chosen action, and agents consciously perceive agency when these predictions are met^37^. Comparator models on action control came to very similar conclusions^46^, as did Haering and Kiesel in their studies on time perception^38^. The prediction of the consequences of voluntary actions is also likely to play a role in IB: predicting a sensory event is likely to speed up its sensory registration, so that predicted stimuli should be perceived earlier than non-predicted stimuli. If thus the selection of a voluntary action is likely to include generating a prediction of the action-related sensory consequences^47^, it is easy to understand why the consequences of voluntary actions are perceived earlier than stimuli following involuntary actions. According to this reasoning, IB would thus increase with the degree of predictability of action effects. If current predictability is high, as in the pilot study, action effects would thus be perceived to follow the action sooner (i.e., IB would be strong). The same may hold if the present predictability is medium but previous predictability was high and smoothly transitioned into medium predictability, as in Experiment 3—so that the present predictability benefits from its successful past. However, this benefit would be eliminated if the transition is too sudden, as in Experiments 1–2. One reason could be that people keep records of ongoing events or situations as long as changes are sufficiently subtle, but reset the record-keeping system in case of more dramatic prediction failures^39,40^. However, while this scenario would account for our finding that the IB pattern obtained in Experiments 1–2 was absent in Experiment 3, it does not quite explain why the pattern reversed. Clarifying this issue will require more research and theorizing. In any case, if our reasoning that IB relies on the degree to which sensory action outcomes can be predicted is sound in principle, IB can indeed be considered to reflect the voluntary nature of the related action even if the size of this effect does not correlate with the degree to which the sensory effects associated with this action are correlated over time—the possibly most relevant source for agency ratings. Accordingly, the lack of correlations between explicit and implicit measures^41^ need not speak to whether or not, or to how reliable they pick up agency-related information. And yet, the lack of such correlations thus tends to undermine the idea that implicit measures of agency, like the IB, can be considered as suitable and equivalent replacements of explicit measures.

We also note that, as compared to the baseline time estimation, the tone onset time in Experiment 1 was perceived as much earlier, while in Experiments 2 and 3 the tone onset time was perceived later and, thus, more like in the baseline condition. We assume the reason is that, in Experiments 2 and 3, the discrimination between previous and present context was not very clear, and thus the predictions of expected action-effects were in a process of changing. This may have caused a feeling of non-exclusivity^37^, in the sense that the virtual hand was controlled by other sources besides participants themselves, which would undermine the sense of agency, and thus the IB effect. This finding is consistent with an earlier study^28^, in which the IB effect was stronger when participants believed that they caused the tone than when they believed the tone was caused by others.

Third, and this brings us to the second aim of our study, there was systematic evidence that the impact of present consistency between one’s own movements and those of the visible artificial body extension on agency was affected by previous consistency experience. With respect to explicit agency judgments, this impact was moderated by the temporal separation between previous and present experience. With a break in between previous and present experience, the former served as a reference against which the latter was matched. This apparently rendered the experience of 50% consistency as rather consistent after having experienced a 0% consistency condition but as rather inconsistent after having experienced a 100% consistency condition. In other words, previous consistency rendered the present explicit and implicit consistency experience relative to the previous experience rather than absolute. Interestingly, this effect reversed in the absence of a temporal separation between the two consistency experiences, irrespective of whether this created a sudden change in consistency, as in Experiment 2, or a rather smooth transition, as in Experiment 3. We take this outcome pattern, together with the observations of Liepelt *et al*.^23^, to imply that previous consistency experience can lead to both assimilation and contrast, depending on the similarity between the context in which consistency is experienced. If this context is or is assumed or perceived to be the same as the context in which the previous consistency experience was made, or if this previous experience is not strongly contextualized anymore (as in the case of a computer mouse or smart phone), assimilation is the likely outcome. If the context is, or is assumed or perceived to be different, as when there is a temporal gap in between previous and present experience, contrast is more likely.

It is interesting to note that this scenario shares some characteristics with Mussweiler’s Selective Accessibility Model (SAM) to social judgment^48^. The model assumes that people firstly engage in a quick holistic assessment of the to be judged individual and some social standard, and then engage in a process of similarity or dissimilarity testing. If testing for similarity, perceived features of similarity will lead to assimilation of individual and standard, whereas encountering features of dissimilarity during dissimilarity testing leads to contrast^49^. In any case, it is important to emphasize that the relationship between previous and present consistency (except for the smoothness manipulation in Experiment 3) affected explicit agency ratings only, but had no impact on IB.

To conclude, our findings provide strong evidence that explicit and implicit indications of human agency integrate various sources of information^10^ that may differ according to circumstances, availability, and (assumed) reliability^8^, and perhaps even to individuals and their sensitivity to particular sources of information^50,51^. In other words, self-related judgments should not be considered readouts of automatically computed, centrally available agency parameters but, rather, as ad-hoc constructions based on the weighted integration of available internal and external cues. Relatedly, our findings also provide converging evidence against the idea that explicit and implicit indicators of agency rely on the same information, which renders the use of implicit indicators as an equivalent of explicit indicators questionable. Our considerations imply that agency is not a natural phenomenon. Rather, agency is a social construction that people can communicate about because they apparently derive similar information from similar informational sources. This might be interesting by pointing to a socially shared practice of information use but is unlikely to reflect a process of basic biological significance.

### Compliance with Ethical Standards

All procedures performed in this study were in accordance with the ethical standards of ethics committee in Southwest University and with the 1964 Helsinki declaration and its later amendments. Informed consents were obtained from all participants included in this study. The experiments were carried out in accordance with the relevant guidelines and regulations approved by the Research Ethical Committee of Southwest University (Chongqing, China).
